# Bibliometric analysis of veterinary medicine on embryo of animals in textbook in conceptualizing disease and health

**DOI:** 10.1016/j.heliyon.2023.e17019

**Published:** 2023-06-05

**Authors:** Maslichah Mafruchati, Wan Iryani Wan Ismail, Akhmad Kusuma Wardhana, Moh. Qudsi Fauzy

**Affiliations:** a Department of Veterinary Anatomy, Faculty of Veterinary Medicine (60115), Universitas Airlangga, Mulyorejo, C Campus, Surabaya, Indonesia; b Faculty of Science and Marine Environment, Universiti Malaysia Terengganu, Malaysia; c Department of Islamic Economy, Faculty of Economic & Business, Universitas Airlangga (60286), Indonesia

**Keywords:** Conceptualization, Genetic diversity and farmed animals, Health and disease, Literacy skills, Veterinary

## Abstract

In veterinary medicine, the concept of disease is critical because it related to the survivability rate of the veterinary, especially livestock. Chicken was the most popular livestock that was observed in veterinary medicine. However, veterinary books were less popular compared to article and conference paper in global academic. This study's goal was to look how was the depiction of topic *disease* was used in veterinary textbooks that were related to the *embryo of chicken* as well as the trend of that topic. This study gathered 90 books meta-data donwloaded from Scopus website in form of CSV file. The data were analyzed using Vosviewer and biblioshiny of R Studio software to see the topic trend, citation, and number of book pages. Literature review also used to see the depiction of *disease* inside samples. Result showed that authors' keywords, *heart and disease* were closely related with a keyword *chicken embryo.* Moreover, each book get at least 10–11 citations globally. Moreover, repetitive keywords used in abstract of samples of this study were *cells/cell, gene,* and *human.* Those repetitive words were closely related to a word *disease.* It could be means that cell of the embryo of chicken also played the important role in determining its resistance against disease.

## 1 Introduction

To acquire a fundamental comprehension of both health and illness, one must take into account veterinary medicine. The veterinary society's increased focus on these fundamental concepts has, however, taken some by surprise [1]. It is crucial to define disease and health in order to recognize diseases because this fundamental distinction between illness and health serves as the foundation for disease classification. Simple explanation of health in veterinary medicine [2].

According to this strict definition of health, being healthy simply means being free from disease. This perspective usually holds for the fundamental theories. The standard assumption in epidemiology is that a disease is binary, for example, an animal either has it or not. This is due to the fact that this is how disease frequency calculations are usually done [3]. Epidemiological techniques, such as, are used to assess the efficacy of a disease test [4].

The sensitivity and specificity of a serological method are based on the implicit assumptions that illness and wellness are concepts with well-defined meanings. It is rare, but not impossible, to evaluate diagnostic techniques using in-depth analysis or more exact definitions [1]. The theoretical concerns surrounding diagnostic tests, for instance, are covered in great detail in a well-known textbook on veterinary epidemiology. Although productivity is frequently used as a substitute for a definition of health in veterinary medicine, Martin and his co-authors note that the concepts of health and disease are only briefly discussed [5].

Veterinary medicine has always valued pathology research. It should go without saying that a pathologist must be able to differentiate between health and disease in order to accurately diagnose a patient [6]. It seems unusual that these fundamental ideas have been explicitly defined or even that the subject is even being researched at all [7]. Anywhere a veterinarian practices, a sizable portion of their regular duties involve dealing with the health and illness of animals, either directly or indirectly. This explains why different disease concepts are used in veterinary medicine and why texts like make sense, such as texts about internal medicine, epidemiology, and animal pathology [8].

The definitions of *disease* in pathology, epidemiology, internal medicine, and other veterinary textbooks are looked at in this study. By using textbooks for veterinary medicine, the terms for *disease* that has correlation with the embryo of chicken was categorized and assessed[9]. Based on the background above this study has a purpose to observe the depiction of concept and term of disease related to embryo of chicken in veterinary books indexed by Scopus. To the best of the authors’ knowledge, there was no previous study which discussed about veterinary books indexed by Scopus related to certain topic. The implications of the various definitions of health are also discussed.

## 2 Methods

### 2.1 Data collection

This study used data about book and book chapter from Scopus website using subscription service. This study used Scopus as the source because Scopus is the eligible international indexing institution of journal and book. The data of indexation of each book and journal is supervised each 3 to 6 months to check if there is any violation of ethical rules of publication. Those who violate the ethic would be discontinue from indexation by Scopus. Moreover, Scopus provided neat and complete meta-data of any journal, paper, or book under the indexation of Scopus.

This study gathered the data through inserting keywords in advanced search feature of scopus website. The keywords were *(title-abs-key (disease) AND title-abs-key (embryo)) AND (chicken) AND (limit-to (doctype, “ch”) OR limit-to (doctype, “bk")).* There were 90 samples of book found. The data were downloaded by clicking several concluded criteria from Scopus such as *citation information, bibliographical information, abstract & keywords.* Aftar those criteria were chosen, the data then were downloaded in to CSV file type. The duration of the study was from 1999 to 2023.

For books on veterinary topics, we combed the libraries. The study made use of veterinary textbooks for pathology, internal medicine, bacteriology, and immunology majors. The index, table of contents, and introductory chapter of each volume were quickly read through to review the literature [10]. A search was done to see if there were any theoretical definitions or explanations of health or disease. We only chose books for further study that provided precise definitions of health and disease[5].

### 2.2 Data analysis

The data were analyzed using bibliometric analysis as well as literature review. For bibliometric analysis this study observe the authors’ keywords trend of certain amount of time, the most used word in abstract, most affiliated county of authors with highest citation, as well as the most book source with highest publication and paper related to disease and health in veterinary field of the study. The software used for bibliometric analysis was R Studio, using its feature, *biblioshiny* [11].

For literature review, this study tried to look up the depiction of health as a topic and a term in veterinary field of study inside book or book chapter used for this study's samples. The data in form of CSV file were observed according from the title and abstract. There were no exclusion of the samples because this paper trie dto look up the depiction of health and disease in the whole samples [12].

## 3 Results

### 3.1 Bibliometric analysis result

**Fig. 1:**
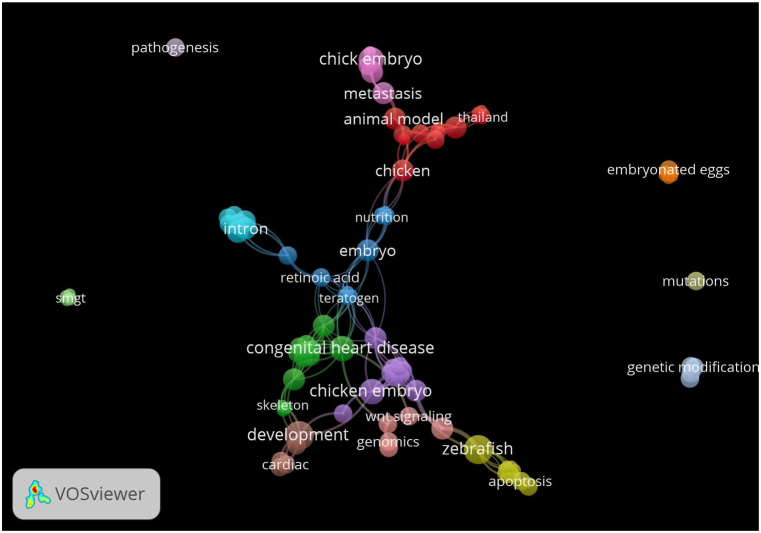
Authors' keywords of veterinary book related to disease in chicken embryo.

**Source:** Generated by Vosviewer 1.1.16

Fig. 1 showed that there was keyword of *disease* depicted in Fig. 1 was closely related to a keyword *chicken embryo, heart,* and *teratogen.* It means that heart disease was the main cause of the mortality of chicken embryo, so that hen needs to be boosted by more vitamins and feeder with more nutritious content to avoid in hatching embryo with weak heart.

**Fig. 2:**
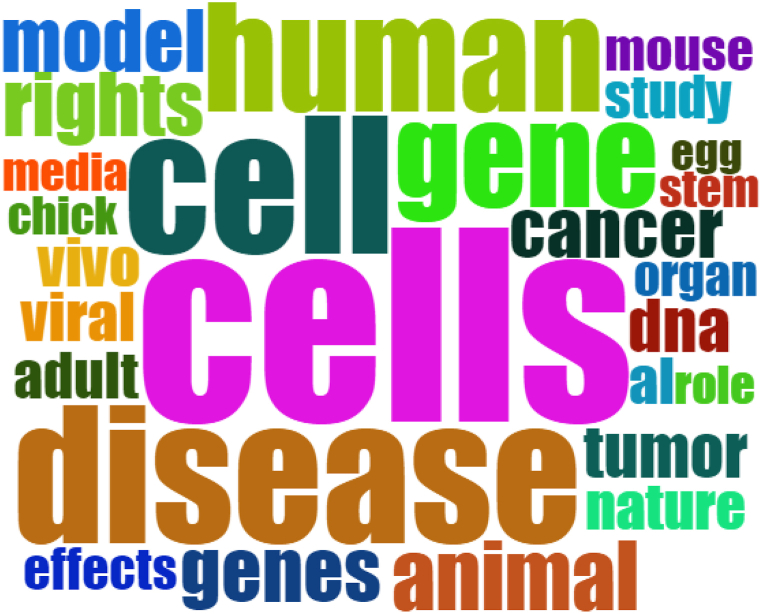
List of frequently used words in abstract related to the disease in embryo of chicken inside samples of this study.

Fig. 2 showed that words *cells/cell, gene,* and *human* were frequently used in abstract of the samples of this study besides a word *disease.* Besides those words, some words that appeared less frequently were *genes, animal, model, rights, tumor, nature,* and *cancer*. Those words appeared less than the previous words but still appeared more than any other words.

**Fig. 3:**
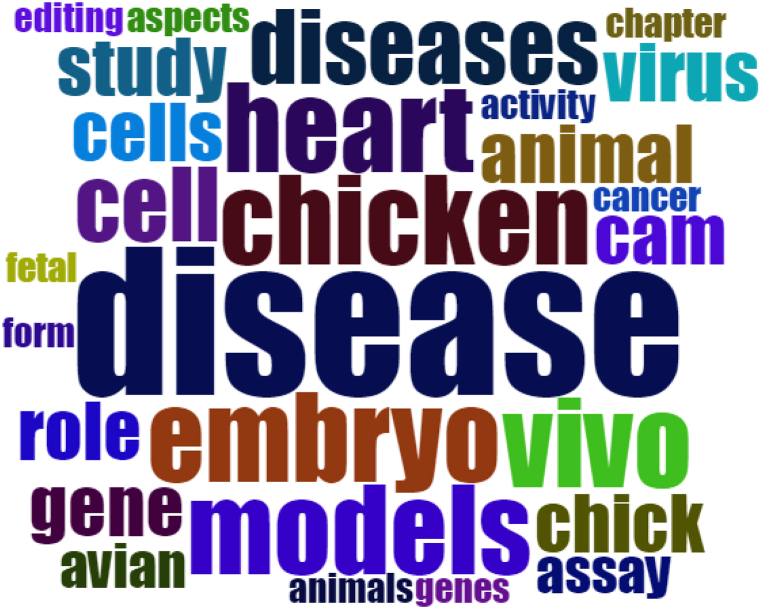
List of frequently used words in title related to the disease in embryo of chicken inside samples of this study.

Fig. 3 showed that words like *embryo, models, vivo, chicken, heart disease, study, cells/cell,* and *animal* were appeared mostly than any other words except a word *disease.*

Table 1 showed that majority of affiliation of authors were from United States of America (USA). USA also the affiliated country with the highest citation with the largest number of books. The second country with highest citation and largest number of book is Japan. It can be concluded that the center of the book publication related to disease of embryo of chicken in the veterinary field of the study is USA. This finding is in line with the study conducted by Ref. [13], where number of book published in an institution would affected to the number of citation received. It is different from the research, review, or conference paper which emphasized on quality content to receive more citation[14].

**Table 1:** List of affiliation of authors with the highest cited veterinary book related to health and disease.

Organization	Documents	Citations
Department Of Ophthalmology And Visual Sciences, University Of Illinois At Chicago, College Of Medicine, Chicago, United States	1	100
Department Of Molecular Biosciences, Faculty Of Life Sciences, Kyoto Sangyo University, Kyoto, Japan	1	95
Embrex, Inc., P.O. Box 13989, Research Triangle Park, 27709, United States	1	85
Department Of Neurosciences, Case Western Reserve University, Cleveland, United States	1	46
Stark Neuroscience Research Institute, Indiana University School Of Medicine, Indianapolis, United States	1	46
Department Of Pediatrics, Northwestern University, Feinberg School Of Medicine, Chicago, United States	1	41
Human Molecular Genetics Program, Children's Memorial Research Center, Northwestern University, Chicago, United States	1	41
Robert H. Lurie Comprehensive Cancer Center, Northwestern University, Feinberg School Of Medicine, Chicago, United States	1	41
University Of Oregon, Institute Of Neuroscience, Eugene, Or, United States	1	40
Research Centers In Minority Institutions, Center For Environmental Health, Jackson State University, Jackson, United States	1	39
Department Of Clinical Chemistry And Laboratory Medicine, University Hospital Carl Gustav Carus Dresden, Dresden University Of Technology, Dresden, Germany	1	35
Institute Of Biology, Leiden University, Leiden, Netherlands	1	35
Program In Reproductive And Adult Endocrinology, National Institute Of Child Health And Human Development, National Institutes Of Health, Bethesda, United States	1	35
Dana Farber Cancer Institute, Howard Hughes Medical Institute, Harvard Stem Cell Institute, Harvard Medical School, 1 Blackfan Circle, Boston, Ma 02115, United States	1	26
Department Of Cell Developmental And Integrative Biology, University Of Alabama At Birmingham, Birmingham, United States	1	26
Division Of Hematology/Oncology, Boston Children's Hospital, Boston, 02115, United States	1	26

Table 2 showed that from 90 books, there were 235 authors. There were 20 single authored docs that were considered as small, compared to the books with collaborative authors. Average citations per documents were 10.51, means that each books could get 10–11 citations.

**Table:** 

Sources	Chapters
METHODS IN MOLECULAR BIOLOGY	17
ADVANCES IN EXPERIMENTAL MEDICINE AND BIOLOGY	6
ENZYMES	5
CURRENT TOPICS IN DEVELOPMENTAL BIOLOGY	4
STRESS AND DEVELOPMENTAL PROGRAMMING OF HEALTH AND DISEASE: BEYOND PHENOMENOLOGY	3
CLINICAL VIROLOGY: THIRD EDITION	2
INTERNATIONAL REVIEW OF CELL AND MOLECULAR BIOLOGY	2
LABORATORY ANIMAL MEDICINE: THIRD EDITION	2
METHODS IN ENZYMOLOGY	2
REPRODUCTIVE AND DEVELOPMENTAL TOXICOLOGY	2
ADVANCED TECHNOLOGIES IN CARDIOVASCULAR BIOENGINEERING	1
ADVANCES IN HEART VALVE BIOMECHANICS: VALVULAR PHYSIOLOGY, MECHANOBIOLOGY, AND BIOENGINEERING	1
ADVANCES IN IMMUNOLOGY	1

**Table 2:** Main information of sample of books used for this study.

Sources (Journals, Books, etc)	55
Documents	90
Annual Growth Rate %	2.93
Document Average Age	6.88
Average citations per doc	10.51
References	1
DOCUMENT CONTENTS	
Keywords Plus (ID)	850
Author's Keywords (DE)	538
AUTHORS	
Authors	235
Authors of single-authored docs	19
AUTHORS COLLABORATION	
Single-authored docs	20
Co-Authors per Doc	2.74
International co-authorships %	13.33

Table 2 showed that the largest number of article as a chapter in a book was 17. The book that have been indexed by Scopus were different fom the ordinary book in terms of the collaboration between authors and purpose of the book. The ordinary books commonly consist of only one or more authors as a content sharing. However, books indexed Scopus was the guide/manual of a specific certain subject of a field of the study. Books indexed by Scopus could also be called as collection of research papers which their length are in terms of number of pages [15].

These books provided a definition of *disease* that could occurred in *embryo of chicken* to give the insight for the academicians and industries to prevent such disease to be happened to their poultries. In addition to providing a definition of health and disease from the perspective of complementary, books that were indexed by Scopus must provide the insight based on the perspective of alternative veterinary medicine [16].

## 4 Discussion

In veterinary textbooks used as samples of this study, there was no any definition of derivative of disease as well as the effect to the embryo of chicken presented as neither either author's keywords nor the frequently used word. Many definitions of health were missing. Most pathology textbooks did not define health and disease. Overall, each organ system was carefully taken into account. There are separate chapters for illnesses of the gastrointestinal tract, the respiratory system, the endocrine system, and so forth. These textbooks included chapters on diseases that affect a particular organ system or a group of diseases, written by various authors. Any category can be used to group definitions of health. However, a division along these lines might facilitate comprehension of concepts involving various definitions of health and disease [22,23].

This study showed that according to Fig. 2, words such as *cells/cell, gene,* and *human* were frequently used in abstract of the samples of this study besides a word *disease.* It could be means that cell of the embryo of chicken also played the important role in determining its resistance against disease. Moreover, cell and gene of the hen and rooster also affected the derivative disease that could be descended to the embryo. Meanwhile, human could avoid the mortality of the embryo of chicken by utilizing more supplement and vitamins to be fed to chickens, so that they would lay eggs that were resistant against disease [17].

Meanwhile, Fig. 3 showed that the chicken embryo model gained interest as the favorite model of the study. Chicken embryo model could be used as immune based study to prevent incoming disease or to be used as biomedical process to be applied in the farm. It was depicted by the frequently used words such as *embryo, models, vivo, chicken, heart disease, study, cells/cell,* and *animal*.

This study also found out that according to the some authors' keywords, words frequently used in abstract, and words frequently used in title, the concept of avoiding *disease* was formulated using two main core or perspective, namely; an adequate state of health, and the biological systems that maintain health. For optimal wellbeing, there must be an explanation of healthy homeostasis when considering the recovery of embryo of chicken from disease.

Other interesting finding was the USA considered as the affiliation of authors written the books related to disease in embryo of chicken with the highest citations. It was not surprising that the center of the veterinary medicine filed, especially related to the poultry was in the USA. Although many countries, especially china and Japan had used the artificial intelligence to control the sanitary and healthiness of their poultries, USA still has becoming a pioneer of publication of research papers in veterinary medicine.

The book with most chapter relevant to the concept and term of *disease* from embryo of chicken was Methods in Molecular Biology. This book is a series under the publisher of *Humana Press* covering research and protocols related to molecular biology. This book could also become the guidance for the veterinary researchers in conducting laboratory research involving animal and its cell culture. Because this book could become the guidance in carrying up the laboratory research, it has higher possibility to get more citation than ordinary theoretical or conceptual books [18].

### 4.1 Literature review

The book entitled *Advances in Immunology* described the definition on *health* is defined as the state of having sound physical and mental condition. Productivity is calculated while taking reproduction into consideration. That book also described about the ailments that affect horses, cattle, sheep, pigs, and goats. A study conducted by Ref. [19] claims that for a long time, subclinical diseases were included in the traditional definition of disease, which is “abnormality of structure or function.

According to a book entitled, *Current Topics in Developmental Biology*, at the anticipated level of nutrient availability and environmental quality is a component of the definition of disease. The definition of a disease will also take into account any lingering drug traces in foods made from animals. In addition to effects on the individual animal, this broader definition of health also takes the environment into account [20].

A book entitled *Avian Immunology* emphasized productivity into consideration in taking care of health of dairy livestocks, with a few minor modifications. The introduction of this book defined *positive health* as the provision of a complete diet. A habitat that satisfies all of the animal's physiological requirements, is pleasing to the eye, offers security and a fear-free environment, is free of pathogenic microorganisms, and is predator- and disease-free [21].

The definition and the one that book entitled *Avian Immunology* suggested for the livestocks were important. By using normative language, the definition illustrated the ideal state of health. Regardless of whether an animal is raised intensively or not, every animal that human raised has a right to be healthy from birth [22,23]. In addition, it went beyond the animal's outward appearance of health, contending that even though an animal may appear to be in good health following treatment for a disease, this does not always imply that it has fully recovered.

It's possible that the main reason for this was the fact that the textbooks' overall goal was to describe diseases, their causes, and their effects rather than to look into what, in general, makes someone healthy or ill. Textbooks used a variety of definitions of health. As a result, it could be more challenging to understand the key health concept as it is presented in a textbook. Textbooks hardly ever include any references to other definitions of health when giving an explicit definition of the concept [24]. This can be explained by the authors' decision to focus on the overall subject of the book rather than a specific health philosophy.

Meanwhile, the book written by Ref. [25] appeared to have written their descriptions of health and disease independently of those provided by other authors, and the general topic of animal welfare was never covered in veterinary textbooks. Several exceptions to this generalization are noted in the texts on veterinary homeopathy. The traditional veterinary medical school is allegedly mechanistic. Definitions of homeopathic health were marketed as being “holistic” and were largely based on the idea that homeostasis controls one's state of health [26].

Moreover, the book entitled *International Review of Cell and Molecular Biology* stated that term *health* was not always necessarily connected to homeopathy because the term *health* in conventional veterinary textbooks may also be based on the homeostasis idea. This finding might imply that veterinary practice is not always impacted by the underlying assumptions of a health definition[27]. It appeared that the author's explicit definition of health related to the embryo of chicken had little to no impact on how the profession viewed both health and disease [22,23]. It would probably be advantageous for veterinary medicine to look more closely at the idea of health. An *animal mechanic* book stated that the idea and concept of *health* was a part of veterinarian who only considers the specific issue the animal is having and fixes it in accordance with the recommended procedures [28].

Communication about these subjects would be improved if terms for health and disease were more clearly defined. The risks posed by zoonoses to the general populace would be higher. The discussion of issues pertaining to animal welfare in general would also become more pertinent by frequently addressing the fundamental ideas of health and illness [29].

## 5 Conclusion

According to the result above, repetitive keywords used in abstract of samples of this study were *cells/cell, gene,* and *human.* Those repetitive words were closely related to a word *disease.* It could be means that cell of the embryo of chicken also played the important role in determining its resistance against disease. Cell mentioned here was the cell of the hen and rooster which determines the resistance of embryo produced against negative microorganism as well as whether an embryo was inherited hereditary disease or not. Since abstract was the core of the paper, they reflected as the most topics that became trend of research by authors. Meanwhile, according to the authors’ keywords, heart disease was the main cause of the mortality of chicken embryo, so that hen needs to be boosted by more vitamins and feeder with more nutritious content to avoid in hatching embryo with weak heart.

According to the literature review, veterinary practice is not always impacted by the underlying assumptions of a health definition. It appeared that the author's explicit definition of health related to the embryo of chicken had little to no impact on how the profession viewed both health and disease. It would probably be advantageous for veterinary medicine to look more closely at the idea of health.

This study limited to only used veterinary books indexed in Scopus as the samples. Further study should be added more books from other indexed institutions along with the other publishers that provided the meta-data of their book to be donwloaded. Thus, it would be beneficial to specify the object of veterinary books discussed, such as poultry, ruminant, freshwater fish, etc.

## Statement of data availability

The underlying data were able to be accessed by public. They were uploaded in Zenodo for the purpose of the research. Link to the dataset is as follows: https://zenodo.org/record/7895516#.ZFNze3ZBx1s.

## Author contribution statement

Maslichah mafruchati: Conceived and designed the experiments; Performed the experiments; Analyzed and interpreted the data.

Wan Iryani Wan Ismail: Performed the experiments.

Akhmad Kusuma Wardhana: Contributed reagents, materials, analysis tools or data.

Mohammad Qudsy Fauzy: Wrote the paper.

## Declaration of competing interest

The authors declare that they have no known competing financial interests or personal relationships that could have appeared to influence the work reported in this paper.
